# The modified Medical Research Council scale for the assessment of dyspnea in daily living in obesity: a pilot study

**DOI:** 10.1186/1471-2466-12-61

**Published:** 2012-10-01

**Authors:** Claire Launois, Coralie Barbe, Eric Bertin, Julie Nardi, Jeanne-Marie Perotin, Sandra Dury, François Lebargy, Gaëtan Deslee

**Affiliations:** 1 Service des Maladies Respiratoires, INSERM UMRS 903, Hôpital Maison Blanche, CHU de Reims, 45 rue Cognacq Jay 51092, Reims, Cedex, France; 2 Unité d'Aide Méthodologique, Pôle Recherche et Innovations, Hôpital Robert Debré, CHU de Reims, Reims, France; 3 Service d’Endocrinologie-Diabétologie-Nutrition, Hôpital Robert Debré, CHU de Reims, Reims, France

**Keywords:** Dyspnea, Obesity, Modified Medical Research Council scale, Six-minute walk test, Lung function

## Abstract

**Background:**

Dyspnea is very frequent in obese subjects. However, its assessment is complex in clinical practice. The modified Medical Research Council scale (mMRC scale) is largely used in the assessment of dyspnea in chronic respiratory diseases, but has not been validated in obesity. The objectives of this study were to evaluate the use of the mMRC scale in the assessment of dyspnea in obese subjects and to analyze its relationships with the 6-minute walk test (6MWT), lung function and biological parameters.

**Methods:**

Forty-five obese subjects (17 M/28 F, BMI: 43 ± 9 kg/m^2^) were included in this pilot study. Dyspnea in daily living was evaluated by the mMRC scale and exertional dyspnea was evaluated by the Borg scale after 6MWT. Pulmonary function tests included spirometry, plethysmography, diffusing capacity of carbon monoxide and arterial blood gases. Fasting blood glucose, total cholesterol, triglyceride, N-terminal pro brain natriuretic peptide, C-reactive protein and hemoglobin levels were analyzed.

**Results:**

Eighty-four percent of patients had a mMRC ≥ 1 and 40% a mMRC ≥ 2. Compared to subjects with no dyspnea (mMRC = 0), a mMRC ≥ 1 was associated with a higher BMI (44 ± 9 vs 36 ± 5 kg/m^2^, p = 0.01), and a lower expiratory reserve volume (ERV) (50 ± 31 *vs* 91 ± 32%, p = 0.004), forced expiratory volume in one second (FEV_1_) (86 ± 17 *vs* 101 ± 16%, p = 0.04) and distance covered in 6MWT (401 ± 107 *vs* 524 ± 72 m, p = 0.007). A mMRC ≥ 2 was associated with a higher Borg score after the 6MWT (4.7 ± 2.5 *vs* 6.5 ± 1.5, p < 0.05).

**Conclusion:**

This study confirms that dyspnea is very frequent in obese subjects. The differences between the “dyspneic” and the “non dyspneic” groups assessed by the mMRC scale for BMI, ERV, FEV_1_ and distance covered in 6MWT suggests that the mMRC scale might be an useful and easy-to-use tool to assess dyspnea in daily living in obese subjects.

## Background

Obesity, defined as a Body Mass Index (BMI) greater than or equal to 30 kg/m^2^, is a significant public health concern. According to the World Health Organization, worldwide obesity has more than doubled since 1980 and in 2008 there were about 1.5 billion overweight adults (25 ≤ BMI < 30 kg/m^2^). Of these, over 200 million men and nearly 300 million women were obese [1].

Dyspnea is very frequent in obese subjects. In a large epidemiological study, 80% of obese patients reported dyspnea after climbing two flights of stairs [2]. In a series of patients with morbid obesity, Collet et al. found that patients with a BMI > 49 kg/m^2^ had more severe dyspnea assessed with BDI (Baseline Dyspnea Index) than obese patients with a BMI ≤ 49 kg/m^2^[3]. The most frequent pulmonary function abnormalities associated with obesity [4,5] are a decrease in expiratory reserve volume (ERV) [6-8], functional residual capacity (FRC) [6-8], and an increase in oxygen consumption [9]. Although the mechanisms of dyspnea in obesity remain unclear, it is moderately correlated with lung function [3,10-16]. Of note, type 2 diabetes [17], insulin resistance [18] and metabolic syndrome [19] have been shown to be associated with reduced lung function in obesity. It must be pointed out that dyspnea is a complex subjective sensation which is difficult to assess in clinical practice. However, there is no specific scale to assess dyspnea in daily living in obesity. The modified Medical Research Council (mMRC) scale is the most commonly used validated scale to assess dyspnea in daily living in chronic respiratory diseases [20-22] but has never been assessed in the context of obesity without a coexisting pulmonary disease.

The objectives of this pilot study were to evaluate the use of the mMRC scale in the assessment of dyspnea in obese subjects and to analyze its relationships with the 6-minute walk distance (6MWD), lung function and biological parameters.

## Methods

### Patients

Adult obese patients from the Department of Nutrition of the University Hospital of Reims (France) were consecutively referred for a systematic respiratory evaluation without specific reason and considered for inclusion in this study. Inclusion criteria were a BMI ≥ 30 kg/m^2^ and an age > 18 year-old. Exclusion criteria were a known coexisting pulmonary or neuromuscular disease or an inability to perform a 6MWT or pulmonary function testing. The study was approved by the Institutional Review Board (IRB) of the University Hospital of Reims, and patient consent was waived.

### Methods

#### Clinical characteristics and mMRC scale

Demographic data (age, sex), BMI, comorbidities, treatments and smoking status were systematically recorded. Dyspnea in daily living was evaluated by the mMRC scale which consists in five statements that describe almost the entire range of dyspnea from none (Grade 0) to almost complete incapacity (Grade 4) (Table 1).

**Table 1:** The modified Medical Research Council (mMRC) scale

**Grade**	**Description of Breathlessness**
Grade 0	I only get breathless with strenuous exercise
Grade 1	I get short of breath when hurrying on level ground or walking up a slight hill
Grade 2	On level ground, I walk slower than people of the same age because of breathlessness, or I have to stop for breath when walking at my own pace on the level
Grade 3	I stop for breath after walking about 100 yards or after a few minutes on level ground
Grade 4	I am too breathless to leave the house or I am breathless when dressing

#### Six-minute walk test

The 6MWT was performed using the methodology specified by the American Thoracic Society (ATS-2002) [23]. The patients were instructed that the objective was to walk as far as possible during 6 minutes. The 6MWT was performed in a flat, long, covered corridor which was 30 meters long, meter-by-meter marked. Heart rate, oxygen saturation and modified Borg scale assessing subjectively the degree of dyspnea graded from 0 to 10, were collected at the beginning and at the end of the 6MWT. When the test was finished, the distance covered was calculated.

#### Pulmonary function tests

Pulmonary function tests (PFTs) included forced expiratory volume in one second (FEV_1_), vital capacity (VC), forced vital capacity (FCV), FEV_1_/VC, functional residual capacity (FRC), expiratory reserve volume (ERV), residual volume (RV), total lung capacity (TLC) and carbon monoxide diffusing capacity of the lung (DLCO) (BodyBox 5500 Medisoft Sorinnes, Belgium). Results were expressed as the percentage of predicted values [24]. Arterial blood gases were measured in the morning in a sitting position.

#### Biological parameters

After 12 hours of fasting, blood glucose, glycated hemoglobin (HbAIc), total cholesterol, triglyceride, N-terminal pro brain natriuretic peptide (NT-pro BNP), C-reactive protein (CRP) and hemoglobin levels were measured.

#### Statistical analysis

Quantitative variables are described as mean ± standard deviation (SD) and qualitative variables as number and percentage. Patients were separated in two groups according to their dyspnea: mMRC = 0 (no dyspnea in daily living) and mMRC ≥ 1 (dyspnea in daily living, ie at least short of breath when hurrying on level ground or walking up a slight hill).

Factors associated with mMRC scale were studied using Wilcoxon, Chi-square or Fisher exact tests. Factors associated with Borg scale were studied using Wilcoxon tests or Pearson’s correlation coefficients. A p value < 0.05 was considered statistically significant. All analysis were performed using SAS version 9.0 (SAS Inc, Cary, NC, USA).

## Results and discussion

### Demographic characteristics

Fifty four consecutive patients with a BMI ≥ 30 kg/m^2^ were considered for inclusion. Of these, 9 patients were excluded because of an inability to perform the 6MWT related to an osteoarticular disorder (n = 2) or because of a diagnosed respiratory disease (n = 7; 5 asthma, 1 hypersensitivity pneumonia and 1 right pleural effusion).

Results of 45 patients were considered in the final analysis. Demographic characteristics of the patients are presented in Table 2. Mean BMI was 43 ± 9 kg/m^2^, with 55% of the patients presenting an extreme obesity (BMI ≥ 40 kg/m^2^, grade 3). Regarding smoking status, 56% of patients were never smokers and 11% were current smokers. The main comorbidities were hypertension (53%), dyslipidemia (40%) and diabetes (36%). Severe obstructive sleep apnea syndrome was present in 16 patients (43%).

**Table 2:** Clinical characteristics of the 45 adult obese patients

	**Total (n = 45)**
**Age**	51 ± 11
**Sex** (M/F)	17/28
**BMI** (kg/m^2^)	43 ± 9
30 ≤ BMI < 35 (kg/m^2^) (grade 1)	7 (16%)
35 ≤ BMI < 40 (kg/m^2^) (grade 2)	13 (29%)
≥ 40 (kg/m^2^) (grade 3)	25 (55%)
**Smoking history**	
Current	5 (11%)
Previous	15 (33%)
Never	25 (56%)
Pack-years	10 ± 17
**Comorbidities**	
Hypertension	24 (53%)
Diabetes	16 (36%)
Dyslipidemia	18 (40%)
Apnea/hypopnea index scores (n/h) (n = 35)	28 ± 20

Data are expressed as mean ± SD or number (%).

### Dyspnea assessment by the mMRC scale and 6MWT

Results of dyspnea assessment are presented in Table 3. Dyspnea symptom assessed by the mMRC scale was very frequent in obese subjects with 84% (n = 38) of patients with a mMRC scale ≥ 1 and 40% (n = 18) of patients with a mMRC scale ≥ 2 (29% mMRC = 2, 9% mMRC = 3 and 2% mMRC = 4).

**Table 3:** Dyspnea assessment of the 45 adult obese patients

	**Total (n = 45)**
mMRC scale (0-4)	1.4 ± 0,9
mMRC scale ≥ 1	38 (84%)
mMRC scale ≥ 2	18 (40%)
Borg scale at rest (1-10)	1 ± 1,5
Borg scale at rest ≥ 1	25 (56%)
Borg scale after 6MWT (1-10)	5.4 ± 2.4
Borg scale after 6MWT ≥ 5	24 (53%)

Data are expressed as mean ± SD or number (%).

mMRC: modified Medical Research Council, 6MWT: six-minute walk test.

The mean distance covered in 6MWT was 420 ± 112 m. Sixteen percent of patients had a decrease > 4% of SpO2 during the 6MWT and one patient had a SpO2 < 90% at the end of the 6MWT (Table 4). The dyspnea sensation at rest was very slight (Borg = 1 ± 1.5) but severe after exertion (Borg = 5.4 ± 2.4). Fifty-three percent of patients exhibited a Borg scale ≥ 5 after the 6MWT which is considered as severe exertional dyspnea. No complication occurred during the 6MWT. Subjects with a mMRC score ≥ 2 had a higher Borg score after the 6MWT than subjects with a mMRC score < 2 (6.5 ± 1.5 *vs* 4.7 ± 2.5, p < 0.05).

**Table 4:** Functional characteristics of the 45 adult obese patients

	**Total (n = 45)**
**Spirometry (n = 45)**	
FEV_1_,% pred	88 ± 18
VC,% pred	92 ± 20
FEV_1_ < 80%	13 (29%)
FEV_1_/VC	0.77 ± 0.10
FEV_1_/ VC < 0.7	5 (11%)
**Plethysmography (n = 38)**	
FRC,% pred	94 ± 23
ERV,% pred	56 ± 34
TLC,% pred	98 ± 17
TLC < 80%	5 (13%)
DLCO,% pred	83 ± 18
DLCO < 70%	10 (26%)
**Arterial blood gases (n = 43)**	
pH	7.42 ± 0,03
PaO2 (mmHg)	90 ± 16
PaO2 ≤ 70 mmHg	0 (0%)
PaCO2 (mmHg)	39 ± 4
PaCO2 > 45 mmHg	0 (0%)
**Walking test (n = 45)**	
6-minute walk distance (m)	420 ± 112
SpO2 at rest (%)	97 ± 2
SpO2 after 6MWT (%)	95 ± 2
Decrease > 4% of SpO2	7 (16%)
SpO2 after 6MWT < 90%	1 (2%)

Data are expressed as mean ± SD or number (%).

FEV_1_: expiratory volume in one second, VC: vital capacity, FRC: functional residual capacity, ERV: expiratory reserve volume, TLC: total lung capacity, DLCO: carbon monoxide diffusing capacity of the lung, pred: predicted value, 6MWT: six-minute walk test.

### Lung function tests

Results of spirometry, plethysmography and arterial blood gases are shown in Table 4. Overall, the PFTs results remained in the normal range for most of the patients, except for ERV predicted values which were lower (ERV = 56 ± 34%). There were an obstructive ventilatory disorder defined by a FEV_1_/VC < 0.7 in 5 patients (11%) with 5 patients (13%) exhibiting a mMRC ≥ 1, a restrictive ventilatory disorder defined by a TLC < 80% in 5 patients (13%) with 5 patients (16%) exhibiting a mMRC ≥ 1, and a decrease in alveolar diffusion defined by DLCO < 70% in 10 patients (26%) with 9 patients (28%) exhibiting a mMRC ≥ 1. Arterial blood gases at rest were in the normal range with no hypoxemia < 70 mmHg and no significant hypercapnia > 45 mmHg.

### Biological parameters

Fifteen percent (n = 7) of patients presented anemia. All patients had a hemoglobin level ≥ 11 g/dL. Mean NT pro-BNP was 117 ± 285 pg/mL. Four patients (10%) had a pro-BNP > 300 pg/mL.Forty-five percent of patients had a fasting glucose level > 7 mmol/L, 51% a Hba1c > 6%, 29% a triglyceride level ≥ 1.7 mmol/L, 35% a total cholesterol level > 5.2 mmol/L and 31% a CRP level > 10 mg/L.

### Relationships between the mMRC scale and clinical characteristics, PFTs and biological parameters

**Figure 1:**
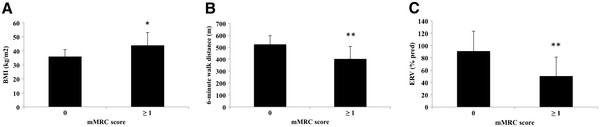
**Differences in Body Mass Index (BMI) (A), Expiratory reserve volume (ERV) (B) and 6-minute walk distance (C) between non-dyspneic (modified Medical Research Council score = 0) and dyspneic (mMRC score ≥ 1) subjects.** *p < 0.05, **p < 0.01. A Wilcoxon test was used.

The comparisons between the mMRC scale and demographic, lung functional and biological parameters are shown in Table 5. Subjects in the mMRC ≥ 1 group had a higher BMI (p = 0.01) (Figure 1A), lower ERV (p < 0.005) (Figure 1B), FEV_1_ (p < 0.05), covered distance in 6MWT (p < 0.01) (Figure 1C) and Hb level (p < 0.05) than subjects in the mMRC = 0 group. Of note, there was no association between the mMRC scale and age, sex, smoking history, arterial blood gases, metabolic parameters and the apnea/hypopnea index.

**Table 5:** Comparisons of patients with mMRC = 0 and patients with mMRC ≥ 1 concerning clinical characteristics, lung function and biological parameters

	**MRC = 0 (n = 7)**	**MRC ≥ 1 (n = 38)**
**Demographic characteristics**		
Age	50 ± 10	51 ± 11
Sex (M/F)	4/3	13/25
**BMI (kg/m**^**2**^**)**	36 ± 5	44 ± 9*
**Smoking history**		
Pack-years	10 ± 16	10 ± 17
**PFTs**		
FEV_1_,% pred	101 ± 16	86 ± 17*
VC,% pred	103 ± 15	90 ± 20
FEV_1_/VC	0.78 ± 0.05	0.77 ± 0.11
FRC,% pred	100 ± 16	91 ± 25
ERV,% pred	91 ± 32	50 ± 31**
TLC,% pred	101 ± 11	97 ± 17
DLCO,% pred	86 ± 18	83 ± 19
**Arterial blood gases**		
PaO2 (mmHg)	99 ± 24	88 ± 14
PaCO2 (mmHg)	39 ± 4	39 ± 4
**6-minute walk test**		
6-minute walk distance (m)	524 ± 72	401 ± 107**
SpO2 at rest (%)	98 ± 2	97 ± 2
SpO2 after exertion (%)	94 ± 2	96 ± 2
Borg score at res	0.1 ± 0.4	1.1 ± 1.6
Borg score after 6MWT	3.9 ± 3	5.7 ± 2.1
**Biological parameters**		
Hemoglobin (g/dL)	14.8 ± 1,3	13.7 ± 1,5*
NT pro-BNP (pg/mL)	97 ± 199	121 ± 301
CRP (mg/L)	5 ± 4,9	9.1 ± 7,4
Triglyceride (mmol/L)	1.9 ± 0,9	1.5 ± 0,8
Total cholesterol (mmol/L)	5.4 ± 1	4.7 ± 1
Fasting glucose (mmol/L)	5.3 ± 0,8	7.8 ± 3,3
Hba1c (%)	5.7 ± 0,6	6.8 ± 1,6

Data are expressed as number (%) or mean ± SD; *p value < 0.05, **p value < 0.01.

FEV_1_: expiratory volume in one second, pred: predicted value, VC: vital capacity, FRC: functional residual capacity, ERV: expiratory reserve volume, TLC: total lung capacity, DLCO: carbon monoxide diffusing capacity of the lung, NT pro-BNP: N-terminal pro brain natriuretic peptide, CRP: serum C reactive protein, HbA1c: glycated hemoglobin.

Normal biological parameters values were based on the normal values for our laboratory: fasting glucose: 3.3 to 6.1 mmol/L; HbA1c: 4 to 6%; total cholesterol: 3 to 5.2 mmol/L; triglycerides: 0.3 to 1.7 mmol/L; NT-pro BNP < 300 pg/mL; CRP < 10 mg/L. Anemia was defined as hemoglobin level < 13 g/dL in men and 12 g/dL in women.

The relationships between the Borg scale after 6MWT and demographic, lung functional and biological parameters were also analysed. The Borg score after 6MWT was correlated with a higher BMI (correlation coefficient = +0.44, p < 0.005) and a lower FEV_1_ (correlation coefficient = -0.33, p < 0.05). No relationship was found between the Borg score after 6MWT and ERV or hemoglobin level. The Borg score after 6MWT was correlated with a higher fasting glucose (correlation coefficient = +0.46, p < 0.005) whereas this parameter was not associated with the mMRC scale (data not shown). We found no statistically different change in Borg scale ratings of dyspnea from rest to the end of the 6MWT between the two groups (p = 0.39).

In this study, 45 consecutive obese subjects were specifically assessed for dyspnea in daily living using the mMRC scale. Our study confirms the high prevalence of dyspnea in daily living in obese subjects [2] with 84% of patients exhibiting a mMRC scale ≥ 1 and 40% a mMRC scale ≥ 2. Interestingly, the presence of dyspnea in daily living (mMRC ≥ 1) was associated with a higher BMI and a lower ERV, FEV_1_, distance covered in 6MWT and hemoglobin level. Furthermore, a mMRC score ≥ 2 in obese subjects was associated with a higher Borg score after the 6MWT (data not shown).

The assessment of dyspnea in clinical practice is difficult. Regarding the mMRC scale, two versions of this scale have been used, one with 5 grades [20] as used in this study and an other with 6 grades [25] leading to some confusion. Other scales have been also used to assess dyspnea [26]. Collet at al. [3], Ofir et al. [11] and El-Gamal [27] et al provided some evidence to support the use of the BDI, Oxygen cost diaphragm (OCD) and Chronic Respiratory Disease Questionnaire (CRQ) to evaluate dyspnea in obesity. El-Gamal et al [27] demonstated the responsiveness of the CRQ in obesity as they did measurements before and after gastroplaty-induced weight loss within the same subjects. The Baseline Dyspnea Index (BDI) uses five grades (0 to 4) for 3 categories, functional impairment, magnitude of task and magnitude of effort with a total score from 0 to 12 [28]. The University of California San Diego Shortness of Breath Questionnaire comprises 24 items assessing dyspnea over the previous week [29]. It must be pointed out that these scores are much more time consuming than the mMRC scale and are difficult to apply in clinical practice.

To our knowledge, the mMRC scale has not been investigated in the assessment of dyspnea in daily living in obese subjects without a coexisting pulmonary disease. The mMRC scale is an unidimensional scale related to activities of daily living which is widely used and well correlated with quality of life in chronic respiratory diseases [20] such as chronic obstructive pulmonary disease (COPD) [21] or idiopathic pulmonary fibrosis [22]. The mMRC scale is easy-to-use and not time consuming, based on five statements describing almost the entire range of dyspnea in daily living. Our study provides evidence for the use of the mMRC scale in the assessment of dyspnea in daily living in obese subjects. Firstly, as expected, our results demonstrate an association between the mMRC scale and the BMI in the comparison between “dyspneic” and “non dyspneic” groups. Secondly, in our between-group comparisons, the mMRC scale was associated with pulmonary functional parameters (lower ERV, FEV_1_ and distance walked in 6MWT) which might be involved in dyspnea in obesity. The reduction in ERV is the most frequent functional respiratory abnormality reported in obesity [6-8]. This decrease is correlated exponentially with BMI and is mainly due to the effect of the abdominal contents on diaphragm position [30]. While the FEV_1_ might be slightly reduced in patients with severe obesity, the FEV_1_/VC is preserved as seen in our study [31]. The determination of the walking distance and the Borg scale using the 6MWT is known to be a simple method to assess the limitations of exercise capacity in chronic respiratory diseases [23]. Two studies have shown a good reproducibility of this test [32,33] but did not investigate the relationships between the 6MWD and dyspnea in daily living. Our study confirms the feasibility of the 6MWD in clinical practice in obesity and demonstrates an association between covered distance in 6MWT and the presence or the absence of dyspnea in daily living assessed by the mMRC scale. It must be pointed out that the 6MWT is not a standardized exercise stimulus. Exercise testing using cycloergometer or the shuttle walking test could be of interest to determine the relationships between the mMRC scale and a standardize exercise stimulus. In our between-group comparisons, BMI and FEV_1_ were associated with the mMRC scale and correlated with the Borg scale after 6MWT. Surprisingly, the ERV was associated with the mMRC scale but not with the Borg scale. Moreover, the fasting glucose was correlated with the Borg scale after 6MWT but not associated with the mMRC scale. Whether these differences are due to a differential involvement of these parameters in dyspnea in daily living and at exercise, or simply related to a low sample size remains to be evaluated.

As type 2 diabetes, insulin resistance, metabolic syndrome [17-19], anemia and cardiac insufficiency have been shown to be associated with lung function and/or dyspnea, we also investigated the relationships between dyspnea in daily living and biological parameters. A mMRC scale ≥ 1 was associated with a lower hemoglobin level. However, all patients had a hemoglobin level > 11 g/dL and the clinical significance of the association between dyspnea in daily living and a mildly lower hemoglobin level has to be interpreted cautiously and remains to be evaluated. Of note, we did not find any associations between the mMRC scale and triglyceride, total cholesterol, fasting glucose, HbA1C, CRP or NT pro-BNP.

The strength of this study includes the assessment of the relationships between the mMRC scale and multidimensional parameters including exertional dyspnea assessed by the Borg score after 6MWT, PFTs and biological parameters. The limitations of this pilot study are as follows. Firstly, the number of patients included is relatively low. This study was monocentric and did not include control groups of overweight and normal weight subjects. Due to the limited number of patients, our study did not allow the analysis sex differences in the perception of dyspnea. Secondly, we did not investigate the relationships between the mMRC scale and other dyspnea scales like the BDI which has been evaluated in obese subjects and demonstrated some correlations with lung function [3]. Thirdly, it would have been interesting to assess the relationships between the mMRC scale and cardio-vascular, neuromuscular and psycho-emotional parameters which might be involved in dyspnea. Assessing the relationships between health related quality of life and dyspnea would also be useful. Finally, fat distribution (eg Waist circumferences or waist/hip ratios) has not been specifically assessed in our study but might be assessed at contributing factor to dyspnea. Despite these limitations, this pilot study suggests that the mMRC scale might be of value in the assessment of dyspnea in obesity and might be used as a dyspnea scale in further larger multicentric studies. It remains to be seen whether it is sensitive to changes with intervention.

## Conclusions

This pilot study investigated the potential use of the mMRC scale in obesity. The differences observed between the “dyspneic” and the “non dyspneic” groups as defined by the mMRC scale with respect to BMI, ERV, FEV_1_ and distance covered in 6MWT suggests that the mMRC scale might be an useful and easy-to-use tool to assess dyspnea in daily living in obese subjects.

## Abbreviations

BMI: Body Mass Index; mMRC scale: Modified Medical Research Council scale; 6MWT: Six-minute walk test; PFTs: Pulmonary function tests; FEV_1_: Expiratory volume in one second; VC: Vital capacity; FVC: Forced vital capacity; FRC: Functional residual capacity; ERV: Expiratory reserve volume; RV: Residual volume; TLC: Total lung capacity; DLCO: Carbon monoxide diffusing capacity of the lung; HbA1c: Glycated hemoglobin; NT pro-BNP: N-terminal pro brain natriuretic peptide; CRP: Serum C reactive protein.

## Competing interests

None of the authors of the present manuscript have a commercial or other association that might pose a conflict of interest.

## Authors’ contributions

CL, CB, EB, JN, JMP, SD, FL and GD conceived the study. CL acquired data. CB performed the statistical analysis. CL and GD drafted the manuscript. All authors read and approved the manuscript prior to submission.

## Pre-publication history

The pre-publication history for this paper can be accessed here:

http://www.biomedcentral.com/1471-2466/12/61/prepub
